# AGEs breaking and antioxidant treatment improves endothelium-dependent dilation without effect on flow-mediated remodeling of resistance arteries in old Zucker diabetic rats

**DOI:** 10.1186/1475-2840-13-55

**Published:** 2014-03-03

**Authors:** Mohamed L Freidja, Emilie Vessières, Bertrand Toutain, Anne-Laure Guihot, Marc-Antoine Custaud, Laurent Loufrani, Céline Fassot, Daniel Henrion

**Affiliations:** 1 INSERM U1083, Angers, France; 2 CNRS UMR 6214, Angers, France; 3 University Hospital (CHU) of Angers, Angers, France; 4 University of Angers, Angers, France; 5 Department of Integrated Neurovascular and Mitochondrial Biology (BNMI), UMR CNRS 6214 - INSERM 1083, Faculté de Médecine, 49045 Angers, FRANCE

## Abstract

**Background:**

A chronic increase in blood flow in resistance arteries is associated with increased lumen diameter (outward remodeling) and improved endothelium (NO)-mediated relaxation. Flow-mediated remodeling of resistance arteries is essential for revascularization in ischemic diseases. Nevertheless, it is impaired in 12 to 24-month old rats and in young Zucker Diabetic Fatty (ZDF) rats due to advanced glycation end products (AGEs) and oxidative stress. As type 2 diabetes occurs preferentially in older subjects we investigated flow-mediated remodeling and the effect of the AGEs breaker ALT-711 associated or not to the antioxidant TEMPOL in one-year old lean (LZ) and ZDF rats.

**Methods:**

Mesenteric resistance arteries were exposed to high (HF) or normal blood flow (NF) in vivo. They were collected after 2 weeks for in vitro analysis.

**Results:**

In LZ rats, diameter expansion did not occur despite a significant increase in blood flow in HF arteries. Nevertheless, endothelium-mediated relaxation was higher in HF than in NF arteries. ALT-711, alone or in combination with TEMPOL, restored outward remodeling in HF arteries in association with AGEs reduction. TEMPOL alone had no effect. ALT-711, TEMPOL or the combination of the 2 drugs did not significantly affect endothelium-mediated relaxation in HF and NF arteries.

In ZDF rats, diameter did not increase despite the increase in blood flow and endothelium-mediated relaxation was further decreased in HF arteries in association with AGEs accumulation and excessive oxidative stress. In both NF and HF arteries, endothelium-mediated relaxation was lower in ZDF than in LZ rats. ALT-711, TEMPOL or their combination did not improve remodeling (diameter equivalent in HF and NF arteries). In parallel, they did not reduce AGEs level and did not improve MMPs activity. Nevertheless, ALT-711 and TEMPOL partly improved endothelium-mediated relaxation through a reduction of oxidative stress and the association of ALT-711 and TEMPOL fully restored relaxation to the level found in LZ rats.

**Conclusions:**

ALT-711 did not improve outward remodeling in mature ZDF rats but it reduced oxidative stress and consequently improved endothelium-dependent relaxation. In mature LZ rats, ALT-711 improved outward remodeling and reduced AGEs level. Consequently, AGEs breaking is differently useful in ageing whether it is associated with diabetes or not.

## Introduction

The frequency of type 2 diabetes increases in most countries so that it is now a major health problem [1] associated with an increased risk of cardiovascular events [2,3]. As the microcirculation provides nutrient and oxygen to distal tissues, damages affecting resistance arteries induce ischemic disorders. Indeed, resistance arteries have a key role in the control of local blood flow. They are able adapt to chronic increases in blood flow, leading to diameter enlargement and improved endothelium (nitric oxide, NO)-dependent relaxation [4]. Flow-mediated outward remodeling of resistance arteries is the driving force for collateral arteries growth following obstruction of a large artery [5,6]. Indeed, after occlusion of a conduit artery, blood flow is redirected through adjacent preexisting collateral vessels, which are consequently submitted to increased shear stress. In contrast to angiogenesis, which is initiated by ischemia, flow-mediated diameter expansion occurs in regions of high-fluid shear stress [7]. Following a chronic increase in shear stress, an inflammatory response associated with oxidative stress occurs leading to activation of matrix metalloproteinases (MMPs) and then by a diameter expansion [8,9].

We have previously shown that outward remodeling of resistance arteries is impaired in young (3-month old) Zucker Diabetic Fatty (ZDF) rats, a model of type 2 diabetes, in combination with a strong reduction in endothelium-mediated dilation [10]. In addition, we found that advanced glycation end products (AGEs) are involved in this dysfunction observed in type 2 diabetic rats as flow-mediated remodeling was recovered in young ZDF rats treated with the AGEs breaker ALT-711 [6]. In 3-month old ZDF rats, AGEs cross-links prevent MMPs activation and induce oxidative stress with increased 3-nitrotyrosine and NADPHoxidase subunit (p67) level [6]. The main limitation of these previous works is that diabetic rats were very young and thus submitted to a very short period of diabetes (a few weeks). AGEs are generated by non-enzymatic glycation of structural proteins by glucose, a process accompanying normal aging and occurring at an accelerated rate in diabetes [11,12]. As the frequency of type 2 diabetes increases with age and vascular damages associated with diabetes develop over time [3] we investigated flow-mediated remodeling in older ZDF rats and assessed the ability of ALT-711, alone or in combination with an antioxidant, to improve outward remodeling and endothelium-mediated dilation in type 2 diabetic rats. We used 12-months old rats, which is the half life expectancy of the non-diabetic rat.

We investigated flow-mediated remodeling using a model allowing the comparison of resistance arteries chronically submitted to high or normal blood flow levels, under similar physiological conditions in the same vascular bed in vivo [6]. ZDF and LZ rats were treated with the AGE-breaker ALT-711, the antioxidant TEMPOL or a combination of ALT-711 and TEMPOL.

## Material and methods

### Animals

**Figure 1:**
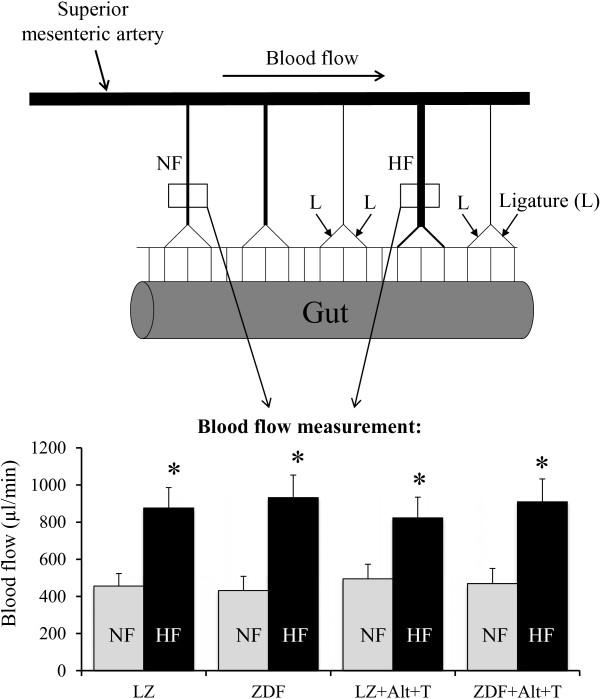
**Experimental model: Schematic representation of the mesenteric circulation in rats and location of the ligations (arrows) of second-order mesenteric artery branches.** The artery located between two ligated vessels was submitted chronically too high flow (HF) artery. Equivalent non-ligated arteries located at a distance from the ligatures had normal flow (NF). Rats were treated with ALT-711 plus TEMPOL (Alt + T) or not. The bargraph shows blood flow measurements obtained using a Transonic ® flow probe in HF and NF arteries of LZ and ZDF rats 14 days after ligation. Values are mean ± SEM (n = 4 to 6 per group). *P < 0.05, HF versus NF.

Twelve-month old adult male ZDF and lean Zucker (LZ) rats (Charles River, L’Arbresles, France) were anesthetized (Isoflurane, 2.5%) and submitted to surgery in order to modify blood flow as previously described [13,14]. Briefly, three consecutive first-order mesenteric arteries were used. Ligatures (7–0 silk surgical thread) were applied to second-order branches of the first and third arteries, as shown in Figure 1. The artery located between two ligated arteries was designated as an HF (high flow) artery. Other arteries located at distance of the ligated arteries were used as control (normal flow, NF) arteries. Rats were treated with buprenorphine (Temgesic®; 0.1 mg/kg, s.c.) before and after surgery. Rats were treated with 4,5-dimethyl-3-phenacylthiozolium chloride (ALT-711, 10 mg/kg per day, ip, n = 10). Treatment started one week before surgery. Another group of rats was treated with the antioxidant 4-hydroxy-2, 2,6,6-tetramethyl piperidinoxyl (TEMPOL, 10 mg/kg per day, gavage, n = 10) and a third group with the combination of ALT-711 and TEMPOL (n = 10). Control groups received the solvents only. The total duration of the treatments was 3 weeks.

Fourteen days after surgery, the rats were anesthetized (Isoflurane, 2.5%) and the right femoral artery catheterized for blood pressure measurement [15]. Blood flow was measured in HF and NF arteries using a Transonic ® flow probe [16]. In short, a medial laparotomy was performed and a section of the ileum was extracted and spread over a gauze swab that had been dampened with a sterile physiological salt solution. The mesenteric artery was dissected free of fat and connective tissue. By use of a micromanipulator, a transit-time ultrasonic flow probe (0.5-mm V series, Transonic Systems) was placed around the artery. Flow was determined with a T106 flow-meter (Transonic Systems). A zero-flow reading was obtained by softly clamping the artery. Then, flow was measured and recorded (Biopac MP100) over a period of 10 minutes (each flow value was the average of at least 3 minutes of recording). Rats were then euthanized by CO_2_ inhalation, the gut excised and the mesenteric arteries gently dissected. HF and NF arteries from each rat were isolated and divided into several segments used respectively for pressure-diameter relationship measurement, pharmacology and biochemistry. Before euthanasia, glycaemia was quantified on a sample of arterial blood using a glucometer [4].

The procedure followed in the care and euthanasia of the study animals complied with European Community Standards on the Care and Use of Laboratory Animals (Ministère de l’Agriculture, France, authorization no. 6422) and the Principles of Laboratory Animal Care (NIH publication no. 85–23, revised 1985; http://grants1.nih.gov/grants/olaw/references/phspol.htm). The protocol was approved by the Committee on the Ethics of Animal Experiments of the “Pays de la Loire” Region (permit # CEEA PdL 2008.10).

### Pressure-diameter relationship in HF and NF arteries

Segments of arteries were cannulated at both ends, mounted on a video-monitored perfusion system (LSI, Burlington, VT) and perfused and superfused with a Ca^2+^-free physiological salt solution (PSS) containing ethylene glycol tetra-acetic acid (EGTA, 2 mmol/L) and sodium nitroprusside (SNP, 10 μmol/L) [17]. Arterial segments were then submitted to a stepwise increase in pressure (10 to 150 mmHg) in order to determine arterial passive diameter [18]. Data were recorded using a Biopac data acquisition system (La Jolla, CA, USA) and analyzed with Acqknowledge® software.

### Pharmacological profile of isolated HF and NF arteries

Other arterial segments (2 mm long) were dissected and mounted on a wire myograph (DMT, Aarhus, DK) [19]. Two tungsten wires (25 μm in diameter) were inserted into the lumen of the arteries and respectively fixed to a force transducer and a micrometer. Arteries were bathed in a PSS of the following composition (mM): 130, NaCl; 15, NaHCO_3_; 3.7, KCl; 1.2 KH_2_PO_4_; 1.2, MgSO_4_; 11, glucose; 1.6, CaCl_2_; and 5, HEPES, pH 7.4, PO_2_ 160 mmHg, PCO_2_ 37 mmHg. Wall tension was applied as previously described [20]. Viability of the arterial segments was tested using a potassium-rich solution (KCl, 80 mmol/L) followed by a concentration-response curve (CRC) to phenylephrine (Phe, 0.001 to 10 μmol/L, 3 min between 2 concentrations, unless a plateau is not reached). A CRC to acetylcholine (ACh, 0.001 to 10 μmol/L, 2 min between 2 concentrations, unless a plateau is not reached) was performed after a precontraction induced by phenylephrine to approximately 50% of the maximal response. Thirty minutes after washout, a second CRC to ACh (0.001 to 10 μmol/L) was performed in the presence of L-NAME (100 μmol/L) or in the presence of superoxide dismutase (120 U/ml) and catalase (80 U/ml). Finally, a CRC to sodium nitroprusside (SNP, 0.001 to 10 μmol/L, 2 min between 2 concentrations, unless a plateau is not reached) was performed.

### Western blot analysis

The remaining segments of NF and HF arteries were used for western-blot analysis. Proteins (25 μg total protein from each sample) were separated by SDS-PAGE using a 4% stacking gel, followed by a 10% running gel. Proteins were detected with specific antibodies (Transduction Laboratories, eNOS 1:1000; AGE 1:100; MnSOD, 1:1000; CuZnSOD, 1:1000; p67phox, 1:1000 and beta-actin 1:1000). Protein expression was visualized using the ECL Plus chemiluminescence kit (Amersham) [21].

### Immunhistological analysis of 3-nitrotyrosine

As previously described [22], segments of arteries were mounted in embedding medium (Tissu-Tek, Miles, Inc), frozen in isopentane pre-cooled in liquid nitrogen, and stored at −80°C. Arterial segments were then pulverised in liquid nitrogen. The powder obtained was resuspended in ice-cold lysis buffer composed of 10 mmol/L Tris–HCl pH 7.4, 1% sodium dodecyl sulfate, 1 mmol/L sodium orthovanadate, and protease inhibitors. Vessel extracts were incubated in this buffer on ice for 30 minutes and then centrifuged (14 000 rpm, 15 minutes, 10°C). The detergent soluble supernatant fractions were retained, and the protein concentration of the samples was determined using a Micro BCA Protein Assay Kit (Pierce). Proteins (15 μg total protein from each sample) were separated by SDS-PAGE and transferred to nitrocellulose membranes. The membranes were incubated with the primary antibody against anti-3-nitro-tyrosine (Upstate and used at a concentration of 20 g/mL) [23]. Fluorescence staining was visualized using confocal microscopy and image analysis (Histolab, Microvision, France) [4].

### In situ zymography

MMPs activity was determined by in situ zymography as previously described [6]. Isolated arterial segments were incubated for 15 min with angiotensin II (100 nmol/L) in PSS. The arteries were then quickly embedded vertically in Tissue-Tek and frozen. The frozen sections (7 μm thick) were incubated over night (37°C) with a fluorogenic gelatin substrate (Molecular Probes) dissolved to 25 μg/mL in zymography buffer (50 mmol/L Tris–HCl, 10 mmol/L CaCl_2_, and protease inhibitor cocktail, pH 7.4) [24]. The gelatin with a fluorescent tag remains caged until the gelatin is cleaved by gelatinase activity. In situ gelatinolysis was revealed by the appearance of fluorescence visualized and quantified using confocal microscopy. In control experiments, sections were incubated with metalloproteinase inhibitors (1,10-phenanthroline and Ethylenediaminetetraacetic acid (EDTA).

### Statistical analysis

Results were expressed as a mean ± SEM. Significance of the variances between groups was determined by a two-way ANOVA followed by the Bonferroni post hoc test. Values of P < 0.05 or lower were considered to be statistically significant.

## Results

**Figure 2:**
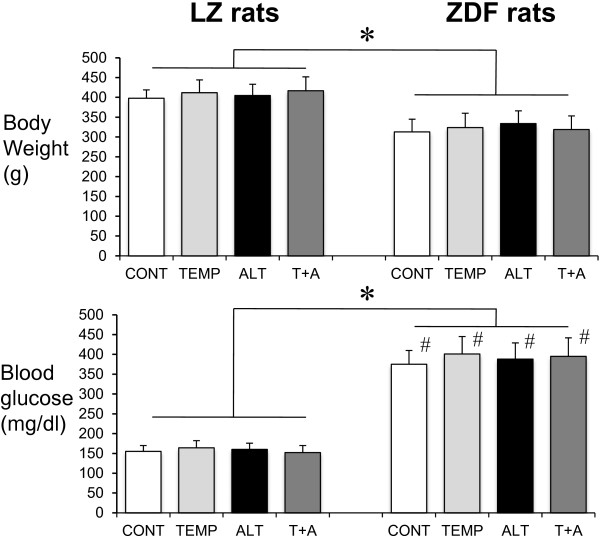
**Body weight and blood glucose in rats: Body weight and blood glucose measured in lean Zucker (LZ) and diabetic fatty Zucker rats (ZDF) in control condition (CONT) or treated with the antioxidant TEMPOL (T), the AGE-breaker ALT-711 (ALT) or the mixture of TEMPOL and ALT-711 (T + A).** Mean ± SEM is presented (n = 10 per group). *P < 0.05, ZDF compared to LZ rats (all treatment groups included). ^#^P < 0.05, ZDF compared to LZ rats (intragroup comparison).

ZDF rats have a significantly lower body weight and greater blood glucose compared to LZ rats (Figure 2).

Blood flow measured in the HF artery was not significantly higher than in the NF artery in both rat strains (Figure 1).

**Figure 3:**
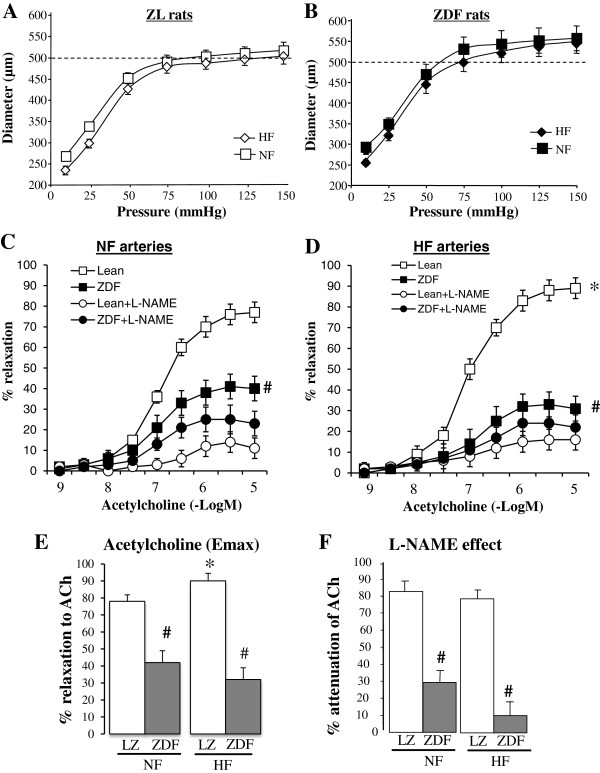
**Flow-mediated remodeling in ZDF and LZ rats: Changes in diameter (A-B) in response to stepwise increases in pressure in HF and NF mesenteric resistance arteries isolated from lean Zucker (LZ, A) and diabetic fatty Zucker rats (ZDF, B).** Concentration-response curves to acetylcholine were performed in NF **(C)** and HF **(D)** arteries in the presence or absence of L-NAME (100 μmol/L). Emax for acetylcholine-mediated relaxation **(E)** and the inhibitory effect of L-NAME on acetylcholine-mediated relaxation **(F)** were calculated from the curves shown in **C** and **D**. Mean ± SEM is presented (n = 10 per group). *P < 0.05, HF arteries compared to NF arteries. ^#^P < 0.05, ZDF compared to LZ.

In both LZ and ZDF rats, the diameter of the HF artery was not significantly different from that of the NF artery (Figure 3A and B). Nevertheless, in LZ rats, endothelium (acetylcholine)-mediated relaxation was greater in HF arteries (Figure 3C) than in NF arteries (Figure 3D) but this was not the case in ZDF rats. This was confirmed by the calculated Emax for acetylcholine-mediated relaxation (Figure 3E). In both NF and HF arteries, acetylcholine-mediated relaxation was lower in ZDF than in LZ rats (Figure 3C-E). In LZ rats, L-NAME reduced acetylcholine-mediated relaxation in both HF and NF vessels, whereas in ZDF rats, L-NAME reduced the relaxation in NF arteries only (Figure 3C). The inhibitory effect of L-NAME was lower in ZDF than in LZ rats in both NF and HF arteries (Figure 3F).

**Figure 4:**
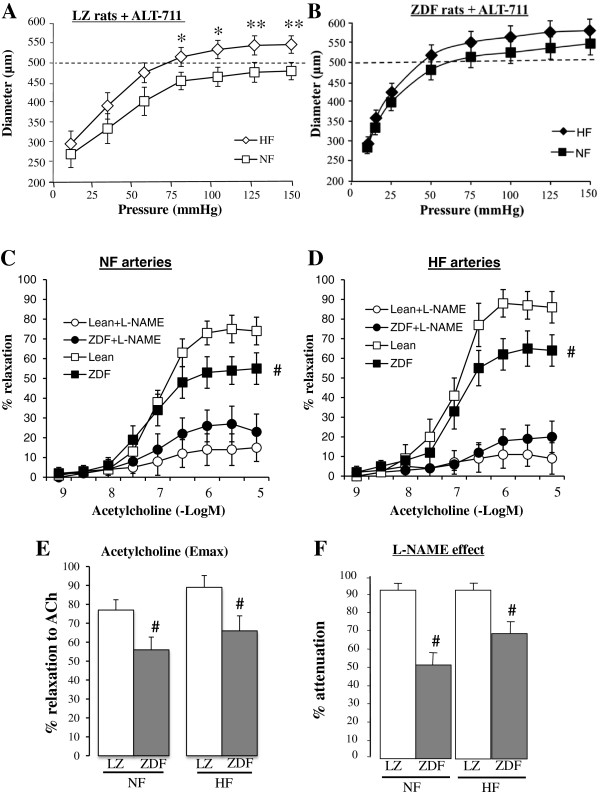
**Flow-mediated remodeling after treatment with ALT-711: Changes in diameter (A,B) in response to stepwise increases in pressure in HF and NF mesenteric resistance arteries isolated from lean Zucker (LZ, A) and diabetic fatty Zucker rats (ZDF, B) treated with ALT-711.** Concentration-response curves to acetylcholine were performed in NF **(C)** and HF **(D)** arteries in the presence or absence of L-NAME (100 μmol/L). Emax for acetylcholine-mediated relaxation **(E)** and the inhibitory effect of L-NAME on acetylcholine-mediated relaxation **(F)** were calculated from the curves shown in **C** and **D**. Mean ± SEM is presented (n = 10 per group). *P < 0.05, HF arteries compared to NF arteries. ^#^P < 0.05, ZDF compared to LZ.

In rats treated with ALT-711 arterial diameter was higher in HF than in NF vessels in LZ rats (Figure 4A) whereas no significant difference was observed between HF and NF arteries in ZDF rats (Figure 4B). Nevertheless, ALT-711 improved acetylcholine-mediated relaxation of NF and HF arteries in ZDF rats (Figure 4C-E). ALT-711 also improved the inhibitory effect of L-NAME in ZDF rats (Figure 4F). ALT-711 had no significant effect on acetylcholine-mediated relaxation and on the the inhibitory effect of L-NAME in NF and HF arteries isolated from LZ rats.

**Figure 5:**
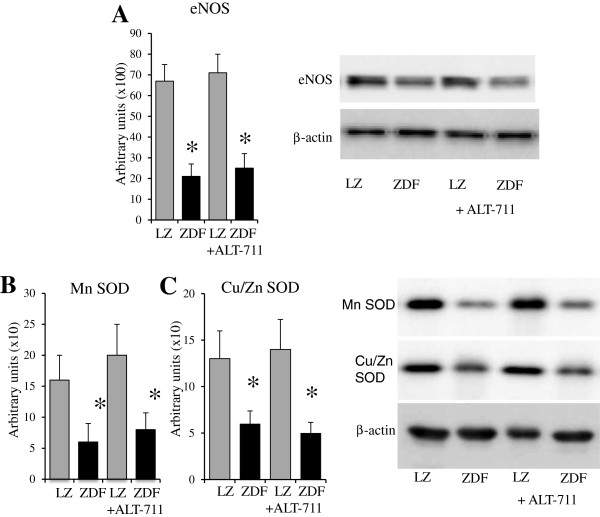
**Measurement of eNOS and SOD in mesenteric arteries: Detection using Western-blot analysis of eNOS (A), MnSOD (B) and Cu/ZnSOD (C)* in mesenteric resistance arteries isolated from lean Zucker (LZ) and diabetic fatty Zucker rats (ZDF) *treated or not with ALT-711. **Mean ± SEM is presented (n = 10 per group). ^*^P < 0.05, ZDF versus LZ.

**Figure 6:**
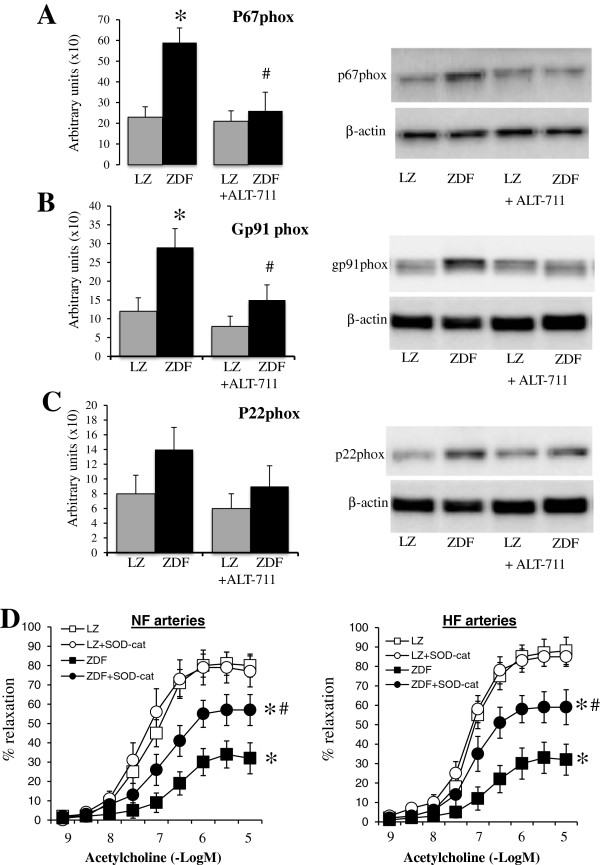
**Detection of NADPH-oxidase subunits in mesenteric arteries: Detection using Western-blot analysis of p67phox (A), gp91phox (B) and p22phox (C) in mesenteric resistance arteries isolated from lean Zucker (LZ) and diabetic fatty Zucker rats (ZDF) treated or not with ALT-711.** The effect of SOD and catalase (SOD-cat) was tested on acetylcholine-induced relaxation **(D)** in NF and HF mesenteric arteries isolated from LZ and ZDF rats. ^*^P < 0.05, ZDF versus LZ. ^#^P < 0.05, ALT-711 compared to control (untreated).

**Figure 7:**
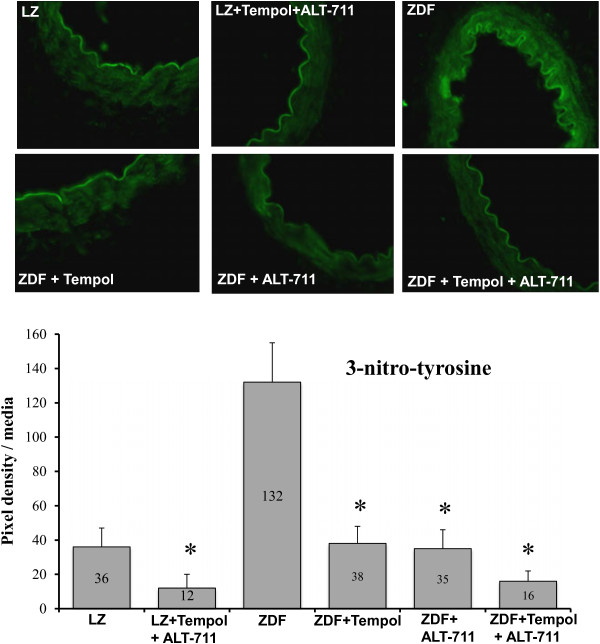
**Detection of 3-nitrotyrosine in mesenteric arteries: 3-nitrotyrosine detection using immunofluorescence was performed in thin sections of NF, or HF arteries using confocal microscopy.** Arteries were isolated from LZ rats or from ZDF rats treated or not with ALT-711, Tempol or Tempol + ALT-711. Image contrast and brightness were increased by 10% in each image to improve visibility. **Bargraph**: Quantification of 3-nitrotyrosine was performed using image density analysis (3–4 arterial sections per artery and 10 rats per group). Mean ± SEM is presented. *P < 0.05, treated versus untreated.

The eNOS, MnSOD and CuZnSOD expression level, reduced in ZDF rats compared to LZ rats, was not affected by ALT-711 (Figure 5). On the other hand, p67phox, gp91phox expression level was higher in ZDF rats compared to LZ rats. ALT-711 reduced it to the same level as in LZ animals (Figure 6A and B). This was not significant for p22phox (Figure 6C). Consequently, we tested functionally the effect of reactive oxygen species on endothelium-dependent relaxation in resistance arteries. Indeed, removing reactive oxygen species acutely using SOD and catalase improved acetylcholine-mediated relaxation in HF and NF vessels isolated from ZDF rats with no change in LZ rats arteries (Figure 6D). These experiments demonstrated that reactive oxygen species reduced acetylcholine-mediated relaxation in arteries of ZDF rats. The effect of ALT-711 on oxidative stress was further confirmed by the measurement of 3-nitro-tyrosine in arteries (Figure 7). 3-nitro-tyrosine was greater in ZDF than in LZ rats. TEMPOL, ALT-711 or their combination significantly reduced 3-nitro-tyrosine level in ZDF rats. Similarly, TEMPOL plus ALT-711 reduced 3-nitro-tyrosine level in LZ rats.

**Figure 8:**
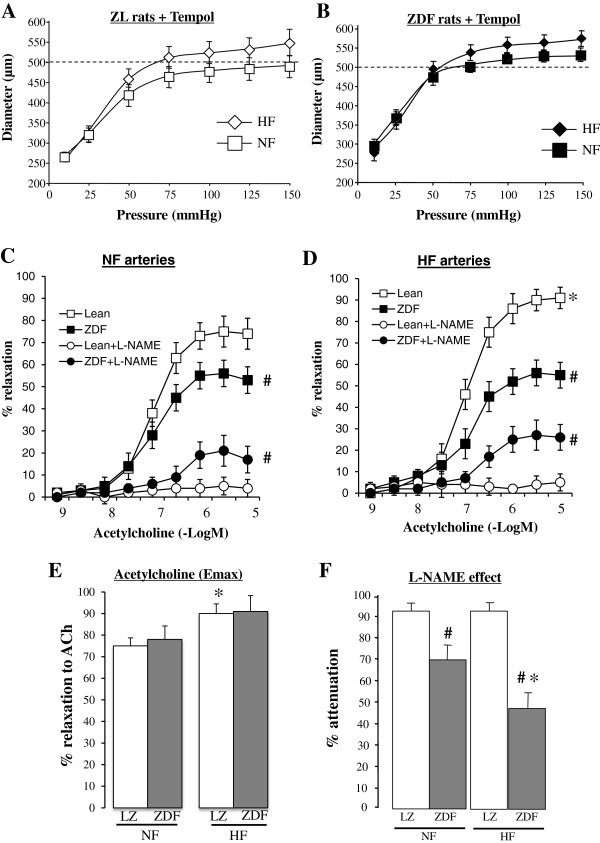
**Flow-mediated remodeling after treatment with TEMPOL: Changes in diameter (A,B) in response to stepwise increases in pressure in HF and NF mesenteric resistance arteries isolated from lean Zucker (LZ, A) and diabetic fatty Zucker rats (ZDF, B**)** treated with TEMPOL.** Concentration-response curves to acetylcholine were performed in NF **(C)** and HF **(D)** arteries in the presence or absence of L-NAME (100 μmol/L). Emax for acetylcholine-mediated relaxation **(E)** and the inhibitory effect of L-NAME on acetylcholine-mediated relaxation **(F)** were calculated from the curves shown in C and D. Mean ± SEM is presented (n = 10 per group). *P < 0.05, HF arteries compared to NF arteries. ^#^P < 0.05, ZDF compared to LZ.

Consequently, ZDF and LZ were treated chronically with the antioxidant TEMPOL. In TEMPOL-treated LZ and ZDF rats, we found no significant difference between HF and NF artery diameter (Figure 8A and B). Acetylcholine-mediated relaxation was higher in HF than in NF arteries in LZ rats, not in ZDF rats (Figure 8C,D and E). L-NAME significantly reduced acetylcholine-mediated relaxation in all groups (Figure 8C,D and F). Thus, TEMPOL improved endothelium (NO)-dependent relaxation in arteries of ZDF rats. This effect was similar to that of ALT711 shown in Figure 4. Nevertheless, by contrast with ALT711, TEMPOL did not improve flow-mediated outward remodeling (no increase in HF arteries diameter). Next, we treated ZDF and LZ rats with a combination of ALT711 and TEMPOL.

**Figure 9:**
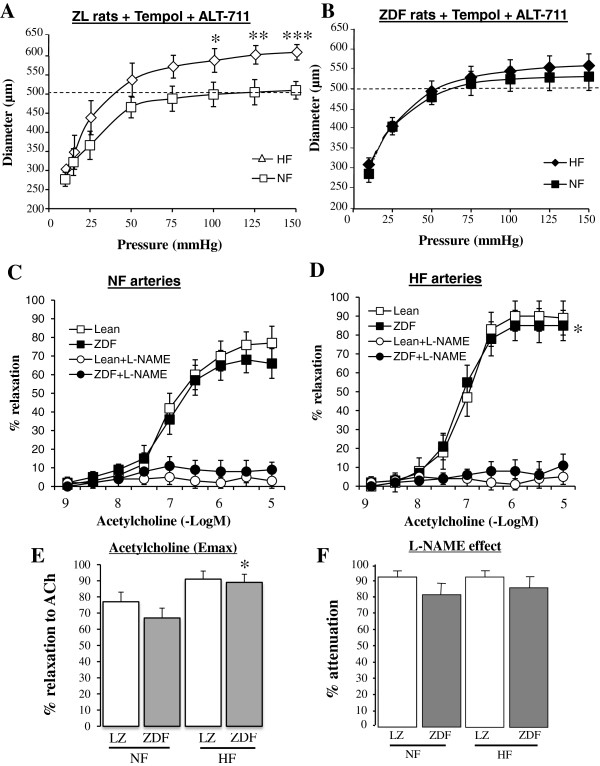
**Flow-mediated remodeling after treatment with ALT-711 and TEMPOL: C hanges in diameter (A,B) in response to stepwise increases in pressure in HF and NF mesenteric resistance arteries isolated from lean Zucker (LZ, A) and diabetic fatty Zucker rats (ZDF, B) treated with TEMPOL and ALT-711.** Concentration-response curves to acetylcholine were performed in NF **(C)** and HF **(D)** arteries in the presence or absence of L-NAME (100 μmol/L). Emax for acetylcholine-mediated relaxation **(E)** and the inhibitory effect of L-NAME on acetylcholine-mediated relaxation **(F)** were calculated from the curves shown in **C** and **D**. Mean ± SEM is presented (n = 10 per group). *P < 0.05, HF arteries compared to NF arteries.

In LZ rats treated with TEMPOL and ALT-711, the HF artery diameter was significantly higher than in NF vessels (Figure 9A). This was not observed in ZDF rats (Figure 9B). Acetylcholine-mediated relaxation was higher in HF than in NF arteries in LZ and in ZDF rats (Figure 9C). Acetylcholine-mediated relaxation was not significantly different in LZ rats compared to ZDF rats (Figure 9C,D and E). L-NAME totally suppressed relaxation in all groups (Figure 9C and D).

**Figure 10:**
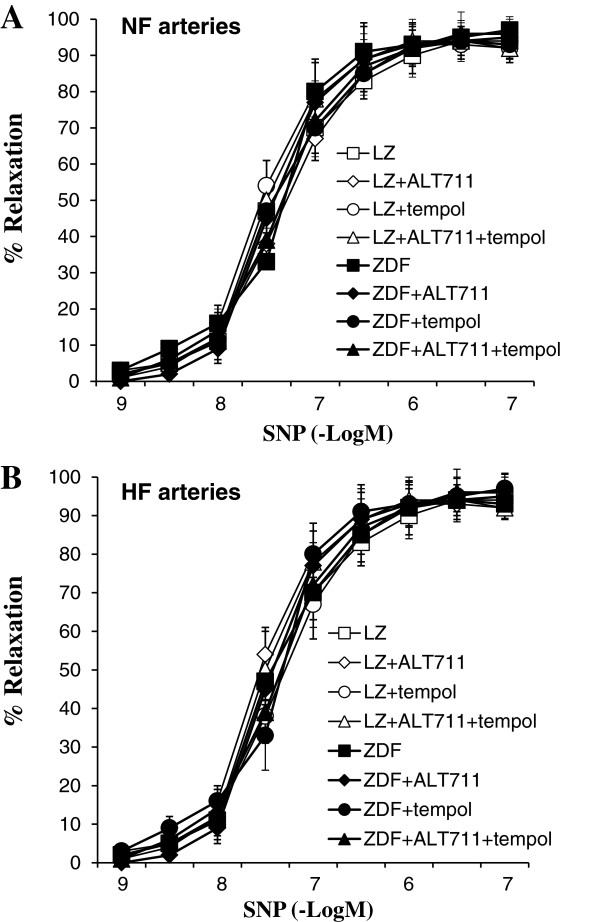
**Vascular response to sodium nitroprusside: Cumulative concentration–response curves to sodium nitroprusside (SNP) obtained in NF (A) and HF (B) arteries isolated from LZ rats or ZDF rats treated or not with ALT-711, TEMPOL or TEMPOL + ALT-711.** Mean ± SEM is presented (n = 10 rats per group).

SNP-mediated dilation, which is independent of the endothelium, was not significantly different in HF and NF arteries in all the study groups. ALT-711 and TEMPOL, alone or in combination, did not alter SNP-mediated dilation (Figure 10).

**Figure 11:**
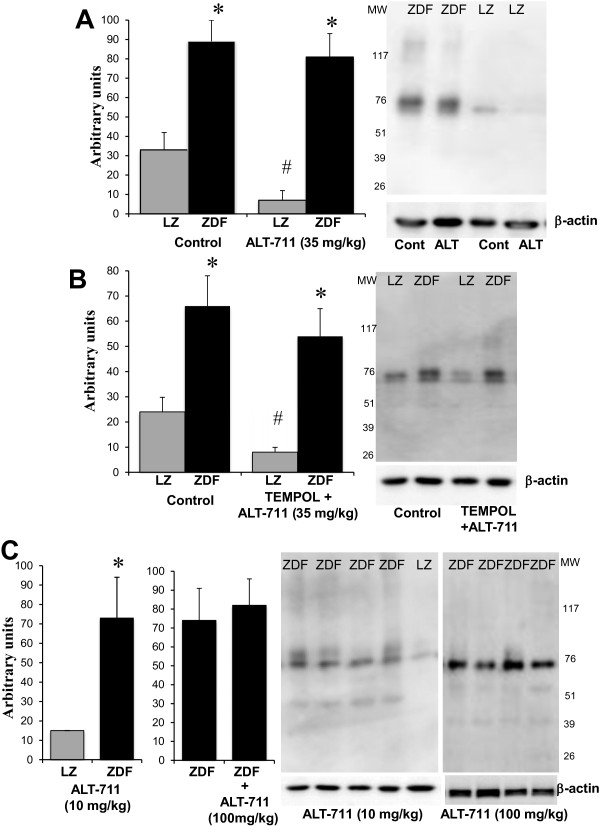
**Detection of advanced glycation end-products in mesenteric arteries: Detection of advanced glycation end-products (AGEs) using Western-blot analysis in mesenteric resistance arteries isolated from ZDF or LZ rats treated with ALT-711 (35 mg/kg per day), ALT-711 (35 mg/kg per day) + TEMPOL (B) or the solvent (control). In another series of experiments (C), rats were treated with ALT-711 (10 mg/kg per day) or ALT-711 (100 mg/kg per day).** Molecular weight (MW) is shown along the blots. Values are presented as mean ± SEM, n = 10 rats per group (panel **A** and **B**). For panel **C** all the measurements are shown. ^*^P < 0.05, ZDF versus LZ. #P < 0.05, treatment versus control.

Advanced glycation end products (AGEs) were higher in ZDF rat arteries than in LZ rat arteries (Figure 11). Nevertheless, ALT-711 alone (data not shown) or in association with TEMPOL did not reduce the level of AGEs in ZDF rats whereas it decreased it significantly in LZ rats (Figure 11).

**Figure 12:**
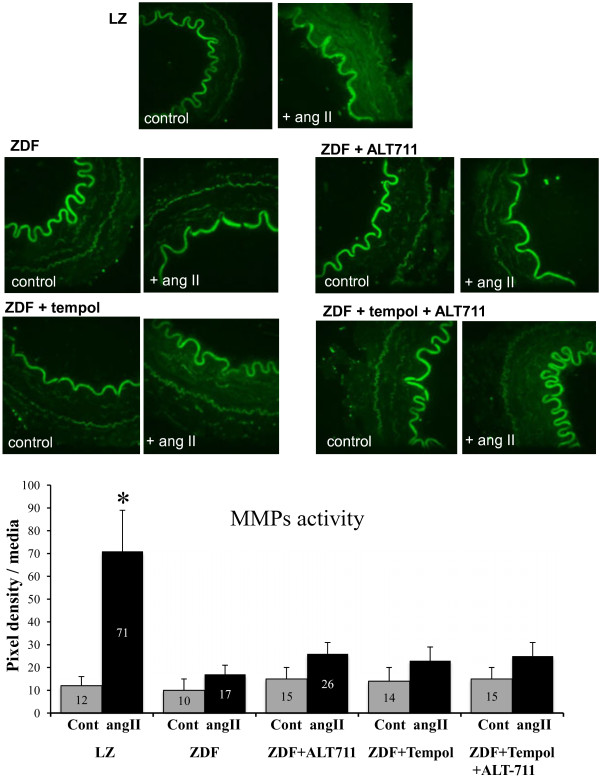
**Measurement of MMPs activity in mesenteric arteries: MMPs activity was measured using in situ zymography performed in arterial thin sections submitted to angiotensin II (angII) or not (control or Cont).** Gelatinase activity was visualized as an enhanced fluorescence of a fluorogenic gelatin substrate in mesenteric arteries using confocal microscopy. Arteries were isolated from LZ rats or from ZDF rats treated or not with ALT-711, TEMPOL or TEMPOL + ALT-711. **Bargraph**: Quantification of gelatinolytic activity was performed using image density analysis (3–4 arterial sections per artery and 6 rats per group). Mean ± SEM is presented. *P < 0.05, Angiotensin II versus control.

As flow-mediated remodeling depends on MMPs activity, we measured MMPs activation in arteries isolated from LZ and ZDF rats. In LZ rats, angiotensin II significantly increased MMPs activity as assessed by in situ zymographic gelatinase activity assay, whereas in ZDF rats angiotensin II induced no significant increase in MMPs activity (Figure 12). The treatment with ALT-711 did not improve the activation of MMPs by angiotensin II. Thus, MMPs activity remained reduced in ZDF rats despite a treatment with ALT711 in association with the antioxidant TEMPOL or not.

## Discussion

In the present study, we found that in one-year old rats, type 2 diabetes impaired not only endothelium-dependent dilation but also the ability of resistance arteries to remodel in response to a chronic increase in blood flow. Ongoing treatment with the AGE-breaker ALT-711 failed to restore flow-mediated remodeling in ZDF rats whereas this treatment improved remodeling in LZ rats. Nevertheless, ALT-711 improved NO-dependent relaxation of mesenteric resistance arteries through reduction of oxidative stress without change in AGEs level.

### Pathophysiology of flow-mediated remodeling

A chronic increase in blood flow (shear stress) in resistance arteries induces outward hypertrophic remodeling and improves endothelium-dependent dilation [4,14,25]. This is essential to adjust organ perfusion in development [26], pregnancy [27], exercise [28] or in response to vasodilator treatments [29]. Furthermore, flow-mediated remodeling of resistance arteries plays a key role in revascularization after occlusion of a large artery as it allows collateral arteries growth and takes part in arteriogenesis [30]. The model used in the present work has the advantage of a vascular bed composed of multiple similar resistance arteries enabling study of the effects of blood flow on the arterial diameter independent of changes in blood pressure or metabolic parameters and without ischemia.

### ALT-711 did not improve flow-mediated remodeling in one-year old ZDF rats

Type 2 diabetes reduced the capacity of the endothelium to induce vasodilatation, especially in resistance arteries [31]. Likewise, in order to restore endothelium-dependent dilation and hopefully local blood flow, vasodilator treatments, drugs improving insulin sensitivity and exercise are commonly recommended to diabetic patients. These treatments and exercise are expected to increase eNOS expression, which is, at least in part, the consequence of the chronic rise in blood flow as shear stress is a major physiological stimulus for eNOS expression [4,25]. In a previous study of mesenteric arteries in young ZDF rats, we have shown that, despite no change in blood glucose level, ALT-711 increased eNOS expression and MMPs activity in association with a strong reduction in AGEs level. All together, these effects improved outward remodeling and endothelium-mediated dilation [6]. In the present study, performed in 12-month-old ZDF rats, ALT-711, alone or combined with the antioxidant TEMPOL, did not improve eNOS expression level, MMPs activity and AGEs level in mesenteric arteries. Consequently, outward remodeling was not improved by ALT-711 in old ZDF rats. This result might not be directly related to age per se because in 12-month-old LZ rats, ALT-711 and ALT-711 plus TEMPOL improved outward remodeling. This is most likely due to the action of ALT-711 on AGEs level as TEMPOL alone had no effect. Furthermore, in 24-month-old non-diabetic rats, hydralazine also improved flow-mediated outward remodeling [32] suggesting that the pathway involved in flow-mediated outward remodeling of resistance arteries remains relatively intact in old rats. A possible explanation is that AGEs cross-links are more stable over time and that ALT-711 cannot reverse preexisting cross-links after long-term exposure to high glucose. Although this assumption remains speculative, several experiments performed in the present study support this hypothesis. Consequently, MMPs activation remained impossible in old ZDF rats and diameter expansion in response to high flow could not take place, although the dose of ALT-711 used in the present study was more than 3-fold greater than in our previous study in younger rats [6]. We also used a dose 10 fold greater in a pilot study (Figure 11) without reducing significantly AGEs level in the vascular wall. In agreement with our finding, in old hypertensive dogs with high level of AGEs, it has been shown that ALT-711 failed to reduce cardiac AGEs contents although it slightly improved cardiac function [33].

### Protective effect of ALT-711 in non-diabetic one-year old rats

On the other hand, in the present study we found that in non-diabetic old rats AGEs content in mesenteric resistance arteries was significantly reduced by the combination of ALT-711 plus TEMPOL. AGEs level was much lower in old LZ rats than in old ZDF rats. In parallel, this combined treatment also improved flow-mediated remodeling in LZ rats. Accordingly, ALT-711 continued to be capable of reducing AGE cross-links in aging and consequently improve vascular remodeling and function as previously shown in large arteries [34,35]. Similarly, ALT-711 therapy in non-diabetic elderly monkeys improves impaired cardiovascular function associated with aging or hypertension [36]. These studies are in agreement with our present work showing that ALT-711 improved outward remodeling of resistance mesenteric arteries in LZ rats with a moderate arterial AGEs level. This is of significant interest as rats were aged one year, which represents half their lifespan. Certainly, the fact that remodeling is lost in 1-year old rats is in agreement with the observations showing that the incidence of cardiovascular events increases in men aged 40 and over or in postmenopausal women [37]. The chronic increase in blood flow restores shear-stress-induced activation of eNOS and antioxidant ability in aged arteries [38] despite an absence of increase in arterial diameter (present study).

### Antioxidant property of ALT-711 and protection of the endothelium

Although it was unable to restore flow-mediated outward remodeling, ALT-711 improved acetylcholine-dependent relaxation in both NF and HF mesenteric resistance arteries in ZDF rats. Although ALT-711 did not affect eNOS expression level, it reduced oxidative stress. Certainly, in ZDF rats, p67phox expression level was increased whereas MnSOD and CuZnSOD levels were decreased. At the same time, in NF and HF arteries in ZDF rats, acute treatment with catalase and SOD improved acetylcholine-mediated relaxation suggesting that oxidative stress contributed mainly to the endothelium dysfunction, in agreement with previous studies [4,39,40]. Certainly, oxidative stress is a major cause of reduced NO bioavailability leading to a reduction in endothelium-mediated relaxation [40]. This was confirmed in the present study using a chronic treatment with TEMPOL, which improved endothelium (NO)-mediated relaxation in ZDF rats, although it remained below control (lean rats) level. The chronic treatment with AL-T711 had a similar effect on endothelium (NO)-mediated relaxation reinforcing the assumption that the beneficial effect of ALT-711 was mediated by an intrinsic antioxidant property. Furthermore, ALT-711 reduced p67phox level in ZDF rat arteries without reduction in AGEs level. The combination of ALT-711 plus TEMPOL totally restored acetylcholine-mediated relaxation to control level. Our observations are in agreement with a work performed by Su *et al.* showing a link between AGEs and oxidative stress in resistance artery endothelial dysfunction in type 2 diabetic mice [12]. Although ALT-711 did not affect AGEs level in arteries isolated of ZDF rats, the present study did not take into account the level of methylglyoxal which is highly increased in diabetes [41] and which has a key role in diabetes-associated endothelial dysfunction. Indeed, excessive methylglyoxal production impairs translocation of glucose transporter 4 trafficking [42] and reduces endothelial NO synthase-associated functions in diabetes [43].

### Physio-pathological consequences

As in young rats, AGEs in old ZDF rats might prevent diameter enlargement in response to a chronic increase in blood flow in addition to inducing endothelial dysfunction. Nevertheless, as the AGE breaker ALT-711, alone or in combination with the antioxidant TEMPOL, failed to reduce the level of AGEs and to restore outward remodeling in old ZDF rats arteries, such a conclusion cannot be directly derived from our data. Nevertheless, we can assume, based on our previous study in young rats [10], that this is probable as the level of AGEs in the present study is higher than in young rats. Interestingly, ALT-711, alone or in combination with TEMPOL improved endothelium (NO)-mediated dilation. This finding may be of importance when recommending exercise or when prescribing vasodilator treatments to diabetic patients. Indeed, both aim to induce flow-mediated outward remodeling and to improve endothelium-mediated dilation [44-46]. Although AGEs-breaking represents an important way of reducing ischemic disorders associated with diabetes, so far preventing their formation remains the best if not the only way, to reduce cross-links in advanced diabetes.

Finally, in one-year-old LZ rats, ALT-711 alone or in association with TEMPOL improved outward remodeling. Accordingly, AGEs-breaking has a potential role in the therapeutic arsenal in cardiovascular diseases in older patients. Improving the response of resistance arteries to shear stress is a key issue in ischemic disorders and whether ageing is associated or not with diabetes AGEs-breaking tools may improve local blood flow due to their effect on outward remodeling and endothelium-mediated dilation.

## Conclusion

We found that the AGE-breaker ALT-711 did not improve outward remodeling in mature ZDF rats although it remained able to reduce oxidative stress and improve endothelium-dependent relaxation. On the other hand, in mature non-diabetic rats, ALT-711 reduced AGEs level and consequently improved outward remodeling. Thus, AGEs breaking, at least using ALT-711, could be a useful therapeutic tool in ageing with antioxidant properties in diabetes and with the capacity to improve outward remodeling in non-diabetic subjects.

## Abbreviations

ACh: Acetylcholine; CRC: Concentration-response curve; NO: Nitric oxide; AGEs: Advanced glycation end products; HF: High blood flow; NF: Normal blood flow; MMPs: Matrix metalloproteinases; Phe: Phenylephrine; SNP: Sodium nitroprusside; ZDF rats: Zucker Diabetic Fatty rats; ALT-711: 4,5-dimethyl-3-phenacylthiozolium chloride; TEMPOL: 4-hydroxy-2, 2,6,6-tetramethyl piperidinoxyl.

## Competing interests

The authors declare that they have no competing interests.

## Authors’ contributions

MLF researched data and performed statistical analyses. EV, BT, ALG researched data and reviewed the manuscript. MAC, LL, CF contributed to discussion, and reviewed the manuscript. DH designed the researched data, wrote the manuscript, obtained financial support. All authors have approved the final version of the manuscript.

## Author information

Daniel Henrion; http://www.bnmi.fr.
